# Can MRI Be Used as a Sensor to Record Neural Activity?

**DOI:** 10.3390/s23031337

**Published:** 2023-01-25

**Authors:** Bradley J. Roth

**Affiliations:** Department of Physics, Oakland University, Rochester, MI 48309, USA; roth@oakland.edu; Tel.: +1-248-375-2703

**Keywords:** artificial intelligence, magnetic resonance imaging, functional MRI, neural activity, biomagnetism, brain, peripheral nerve, heart, Lorentz force, phase shift

## Abstract

Magnetic resonance provides exquisite anatomical images and functional MRI monitors physiological activity by recording blood oxygenation. This review attempts to answer the following question: Can MRI be used as a sensor to directly record neural behavior? It considers MRI sensing of electrical activity in the heart and in peripheral nerves before turning to the central topic: recording of brain activity. The primary hypothesis is that bioelectric current produced by a nerve or muscle creates a magnetic field that influences the magnetic resonance signal, although other mechanisms for detection are also considered. Recent studies have provided evidence that using MRI to sense neural activity is possible under ideal conditions. Whether it can be used routinely to provide functional information about brain processes in people remains an open question. The review concludes with a survey of artificial intelligence techniques that have been applied to functional MRI and may be appropriate for MRI sensing of neural activity.

## 1. Introduction

In 1984, J. H. Nagel submitted a three-sentence abstract to the Sixth Annual Conference of the IEEE Engineering in Medicine and Biology Society titled “NMR Imaging of Action Currents” [1].

“The magnetic field that is generated by action currents is used as a gradient field in NMR imaging. Thus, the bioelectric sources turn out to be accessible inside the human body while using only externally fitted induction coils. Two- or three-dimensional pictures of the body’s state of excitation can be displayed.”

Did Nagel really use nuclear magnetic resonance (NMR)—now called magnetic resonance imaging (MRI)—to sense action currents? Probably not. As shown in this review, employing MRI as a sensor of neural activity is challenging. Technology available 40 years ago was probably not up to the task. Yet, Nagel’s idea of using MRI to detect bioelectricity has become an active and growing field of study that could eventually revolutionize our ability to monitor the brain.

Researchers have been measuring electrical current using MRI for decades. Michael Joy and his colleagues showed that they could measure the magnetic field in the body using MRI, take the curl, and determine the current density [2,3,4]. They developed this method, but to image *applied* currents, such as those for stimulating nerves or defibrillating the heart. Bioelectrical currents produced *within* the body itself are usually smaller and more difficult to detect.

Magnetic resonance can monitor brain activity using a method called functional MRI (fMRI) [5,6]. This technique detects changes in blood flow to metabolically active neurons instead of neural electrical activity. Therefore, this “blood-oxygen-level-dependent” (BOLD) mechanism measures the brain’s activity indirectly. Moreover, fMRI signals cannot follow the millisecond dynamics associated with action potentials. This review examines studies that have attempted to go beyond fMRI to directly sense action potentials.

The direct detection of brain activity using MRI would have many uses. As a research tool, it could monitor phenomena such as perception, memory, and brain plasticity. As a clinical tool, it could help diagnose stroke, detect disorders such as Parkinson’s disease, and assist in pre-surgical planning by localizing pathologies such as tumors. As a psychological tool, it could assist in identifying mental illnesses such as schizophrenia, anxiety, autism, obsessive-compulsive disorder, and attention deficit/hyperactivity disorder. Functional MRI has been used for all these applications and direct recording of neural currents using MRI should have uses similar to fMRI, but with better temporal resolution and less uncertainty arising from fMRI’s indirect detection of blood flow rather than brain electrical activity. Electroencephalography and magnetoencephalography are also used for monitoring brain activity, but they require solving a difficult and nonunique inverse problem to localize the source, which would not be required for direct MRI measurement of neural current.

Nearly twenty years ago, Peter Bandettini, Natalia Petridou, and Jerzy Bodurka analyzed direct detection of neuronal activity with MRI, asking if it was “fantasy, possibility, or reality” [7]. This question has still not been definitively answered. Recent studies suggest that “fantasy” is too pessimistic. “Possibility” seems a better description of the current opinion, while “reality”—in the sense of having an established technique to routinely record brain activity—is not available yet, although it may come about in the future.

## 2. MRI Measurement of Activity in the Heart

Although monitoring the brain is the ultimate goal of most research into using MRI as a sensor of electrical activity, the strongest source of bioelectrical current in our bodies is the heart, so it is a reasonable place to begin this review. The first task is to estimate the size of the magnetic field produced inside the heart by action potential wavefronts propagating through the cardiac tissue. The magnetocardiogram measures the magnetic field produced by the heart and often records a signal with a strength of several picotesla (pico = 10^−12^). These signals, however, are detected outside the body. What is the size of the magnetic field inside the heart?

**Figure 1:**
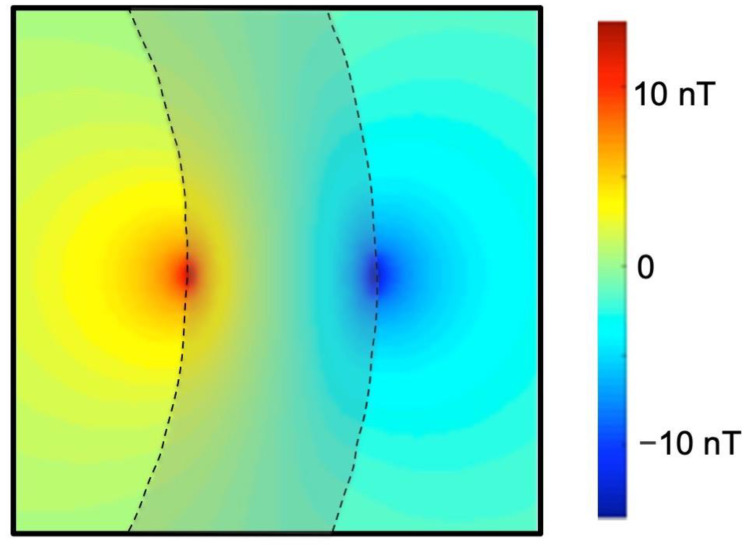
The magnetic field produced by an action potential wavefront in the heart. The heart is spherical and only a portion of the heart wall is shown (shaded, 1 cm thick). On the left is part of the blood cavity enclosed by the heart and on the right is part of the bath surrounding the heart. A wavefront propagates from top to bottom, with the upstroke positioned halfway down. Red corresponds to the magnetic field directed into the paper and blue out of the paper. Adapted from Figure 5 of [8].

Dan Xu and Brad Roth calculated the magnetic field produced in the heart using the bidomain model (a mathematical model of cardiac tissue that accounts for the intracellular and interstitial spaces separately) [8]. They found that the magnetic field is largest at the surface of the heart wall (Figure 1) and has a size of about 10 nanotesla (nano = 10^−9^). As will become clear in this review, the nanotesla (nT) is the natural unit to use when discussing biomagnetic fields in the body. Such fields are a billion times weaker than the static magnetic fields used in modern magnetic resonance imaging devices. This calculation highlights one of the key challenges when using MRI as a sensor of action currents: the magnetic field produced by those currents is tiny.

Xu and Roth’s study also indicates the spatial and temporal resolution needed to image electrical activity in the heart. The upstroke of the cardiac action potential lasts only about half a millisecond, which is significantly shorter than the time required to obtain even one slice of a magnetic resonance image. The wavefront’s propagation speed is on the order of 0.5 m/s, implying that the spatial extent of the depolarization phase of the action potential extends over a few tenths of a millimeter. Without sub-millimeter spatial resolution, any MRI detection will suffer from partial volume averaging.

Magnetic resonance imaging works through changing the precession of magnetic spins—usually hydrogen nuclei in water—by varying the magnetic field. The precession frequency is set primarily by the strong static magnetic field of the MRI machine, which is typically a few tesla. However, the frequency and phase of the spin precession is modified by any other magnetic field that is present, including the magnetic field gradients used in an MRI pulse sequence for imaging. As Nagel noted, the biomagnetic field associated with action currents will also shift the phase of the spins and can thus serve as a type of gradient field when imaging. This phase shift is the product of the magnetic field strength, the time for which it is present, and the spin’s gyromagnetic ratio (for hydrogen, 2.7 × 10^8^ radians/s/T). If the cardiac magnetic field in the heart produces a 10 nT field lasting 0.5 ms, the phase shift will be about 0.001 radians, or roughly a tenth of a degree. The MRI technology needs to be able to detect phase shifts of this size in order to sense cardiac electrical activity.

Why have no experimental studies been performed to use MRI as a sensor for wavefronts in the heart? Almost certainly the reason is motion artifacts. The purpose of the heart is to pump blood, for which it must contract. The resulting motion of tens of millimeters affects the MRI signal much more than does the miniscule magnetic field perturbations associated with action currents. Magnetic resonance images of the heart are sometimes obtained by gating the pulse sequence to the electrocardiogram, but such gating would need to be extraordinarily precise to prevent all motion artifacts. If employed as a research instrument rather than a clinical tool, one could try using MRI to study biocurrents in a Langendorff-perfused heart in which motion has been suppressed by a drug to block excitation–contraction coupling. However, the drug would have to abolish even microscopic motion in order to make direct detection of biomagnetic fields feasible. In general, the movement of the heart makes MRI-based recordings of cardiac action currents difficult or impossible.

## 3. MRI Measurement of Activity in Peripheral Nerves

### 3.1. The Magnetic Field Produced by a Peripheral Nerve

**Figure 2:**
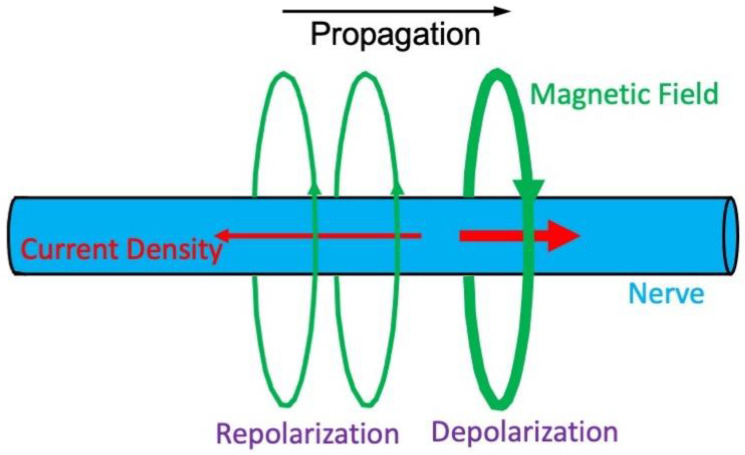
The magnetic field produced by an action potential in a peripheral nerve [9,10]. The action potential propagates from left to right down the cylindrical nerve (blue). Current (red) associated with the depolarization phase of the action potential points in the direction of propagation and current associated with the repolarization phase points opposite to the direction of propagation. The magnetic field (green) wraps around the action current.

The magnetic field produced by a peripheral nerve has been measured (Figure 2) [9,10]. Ranjith Wijesinghe and Roth used these studies to estimate the biomagnetic field and MRI phase shift produced in diverse situations [11]. For instance, the magnetic field just outside a squid giant axon is 2.4 nT. The lower value compared with that in the heart is primarily because nerve action potentials have faster propagation speeds, so the action potential upstroke is spread over a longer distance, thereby reducing the current density along the axon. The squid axon’s upstroke is similar to (and perhaps slightly slower than) the heart’s. These considerations led Wijesinghe and Roth to conclude that the phase shift in the MRI signal from a peripheral nerve is about 0.015°. The phase shifts associated with a frog sciatic nerve, a human median nerve, and a rat extensor digitorum longus muscle should be even smaller. Based on their analysis, Wijesinghe and Roth were pessimistic about being able to detect these fields, concluding that “MRI measurements of action current in nerve and muscle are unlikely using current technology” [11].

**Figure 3:**
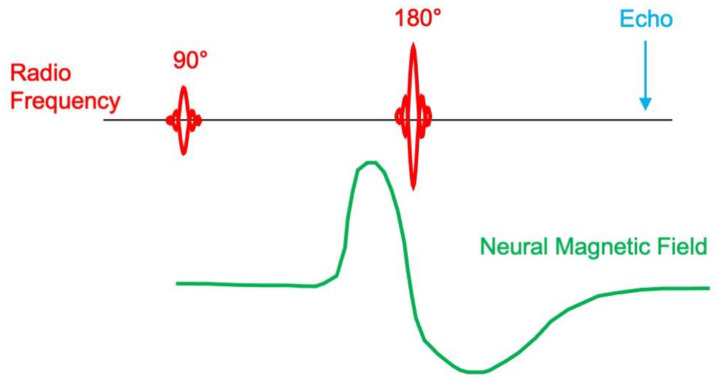
The optimum timing of the neural magnetic field waveform during a spin-echo pulse sequence. The initial depolarization (positive) phase of the signal occurs before the 180° radiofrequency pulse, and the repolarization (negative) phase occurs after it. In this case, the phase shifts all combine to produce the largest effect on the echo. Adapted from Figure 1 of [7].

Bandettini et al. note that the timing of the magnetic field is crucial [7]. If the entire action potential (both depolarization and repolarization phases) happens in the time between the 90° and 180° pulses of a spin-echo pulse sequence, then there is no net phase shift and the echo looks like it would if no biomagnetic field were present. Similarly, if the entire action potential happens between the 180° pulse and the echo, then it would have no effect. The neural response will be largest if the depolarization phase of the action potential is before the 180° pulse and the opposite polarity repolarization phase is after the 180° pulse (Figure 3), in which case the phase shifts combine and the echo is affected. This demonstrates how sensitive the technique is to the exact timing of the neural magnetic field.

In 2009, Martyn Paley and his coworkers tried to use MRI to sense action potentials in the optic nerve [12]. They were unable to record a signal and found “no clear evidence for direct detection [of the biomagnetic field] in these experiments”. They point out the same concern that Bandettini et al. [7] raised: the nerve action potential is biphasic (Figure 3) and lasts only a few milliseconds, so the phase shifts associated with depolarization and repolarization might cancel out in the MRI recording. This is not a significant problem in the heart, where the repolarization phase of the action potential occurs over hundreds of milliseconds.

Paley et al. used “ghost reconstructed alternating current estimation” (GRACE) to search for a signal [12,13]. The idea is that a weak magnetic field caused by an alternating current produces ghost artifacts on magnetic resonance images. They first applied hundreds of microamps of current (producing magnetic fields of tens of nanotesla) through phantom coils to mimic neural activity and were able to detect the ghost images. However, when they presented an oscillating visual stimulus to the eye of a healthy volunteer, they could not detect any ghosts.

### 3.2. Lorentz Force Imaging

**Figure 4:**
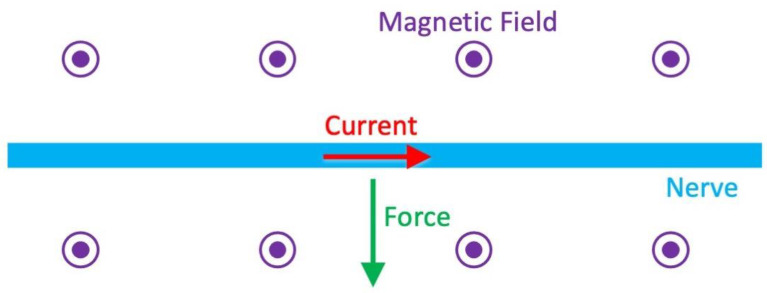
The Lorentz force acting on a nerve. The magnetic field (purple) is uniform and directed out of the paper. An action potential propagates from left to right along a nerve (blue). The action current associated with the upstroke of the action potential (red) experiences a magnetic force (green) according to the “right-hand-rule”; this Lorentz force is perpendicular to both the current and the magnetic field.

Allen Song, Trong-Kha Truong, and their collaborators have proposed another technique for recording action currents in peripheral nerves, based on “Lorentz effect imaging” [14,15,16]. When exposed to a strong static magnetic field, like that in an MRI device, neural action currents are subjected to a magnetic force, often called a Lorentz force, that moves the nerve (Figure 4). If this motion is large enough, it produces an artifact in the magnetic resonance image that can be used to detect action currents.

**Figure 5:**
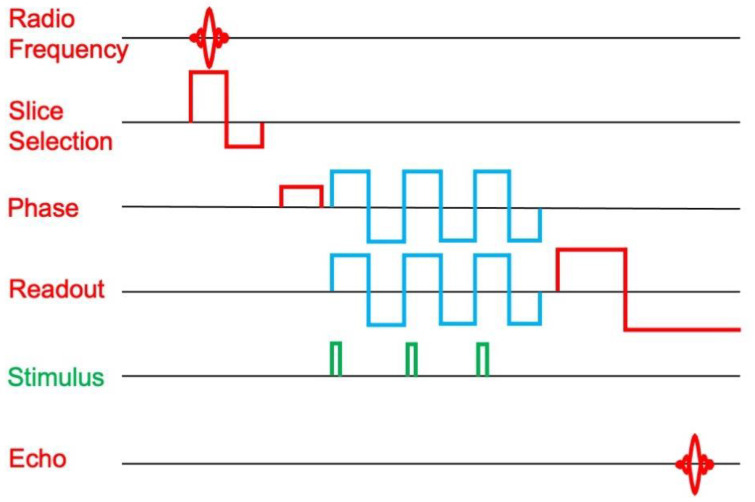
A traditional MRI gradient-echo pulse sequence with slice selection, phase encoding, and readout gradients (red). Truong et al. [16] applied additional oscillating gradients (blue) in synchrony with electrical stimulation of the median nerve at the wrist (green). Ideally, depolarization of the nerve action potential occurs during the positive phase of the oscillating gradient and repolarization during the negative phase. In that case, the echo is slightly different depending on whether or not the nerve is stimulated.

Their hypothesis is that motion of the nerve will cause dephasing of the spins, lowering the MRI signal. They amplify this effect by applying oscillating magnetic field gradients in synchrony with the neural stimulation (Figure 5). They claimed to see an MRI signal associated with stimulation of the median nerve at the wrist [16].

**Figure 6:**
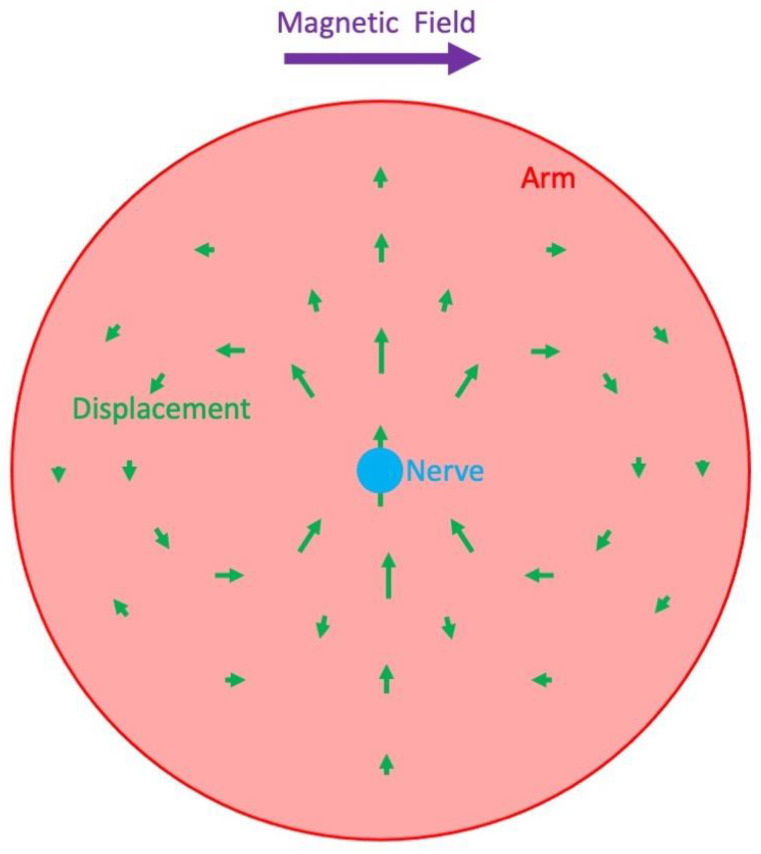
The displacement caused by the Lorentz force acting on a nerve in a magnetic field. The nerve (blue) is shown in cross section and is centered in an arm (red). It carries an action current that is directed out of the paper. The magnetic field (purple) is to the right. The displacement arrows (green) are exaggerated in this illustration to make them easier to see. Adapted from Figure 2 in [17].

Is the motion of the nerve big enough to be detectable? Roth and Peter Basser calculated the nerve displacement [17]. The magnetic force was estimated as the current density times the magnetic field strength (they used 4 T), and the elastic restoring force was determined from the shear modulus of the surrounding tissue. They predicted that, for realistic parameter values, the displacement of the nerve is about 10 nm (more than a hundred times less than the diameter of a single axon and nearly a million times less than the diameter of the median nerve). In addition, the displacement is widespread throughout the tissue rather than being localized near the nerve (Figure 6). They concluded that such inconsequential, diffuse displacements would not be detectable using conventional MRI. To make matters worse, Roth and Basser suspected that they overestimated the displacement because they assumed the entire median nerve was simultaneously active [17]. Yet, Truong and Song stimulated the nerve below the motor fiber threshold to avoid any motion artifact, so only low-threshold sensory fibers were activated [16]. 

Roth and Basser were not able to determine what mechanism was responsible for Truong and Song’s observation, but they estimate that the effect from the biomagnetic field should be much greater than the effect caused by magnetically induced displacement. The oscillating gradients should act similar to the gradients used to measure diffusion with MRI, so diffusion artifacts might be responsible for part of the signal. In addition, slight muscle contractions caused by the inadvertent stimulation of some motor axons could produce small motions that are nevertheless many times larger than that produced by the Lorentz force. More sophisticated calculations that required fewer assumptions support Roth and Basser’s conclusion that the motion of a nerve caused by the Lorentz force is too small to be measured with MRI [18].

**Figure 7:**
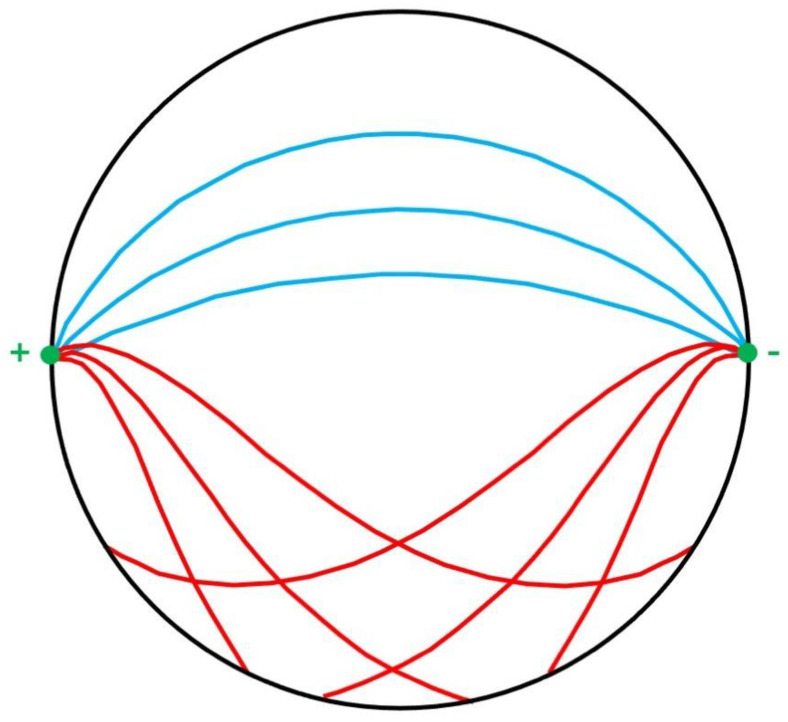
The trajectories of copper and sulfate ions in water. The magnetic field is directed out of the paper. Blue: calculated using realistic ion mobilities (the trajectories are almost the same as those when the magnetic field is zero). Red: calculated using Truong et al.’s ion mobilities [19]. Adapted from Figures 1 and 2 in [20].

Truong, Avram, and Song extended their study of this technique by analyzing the Lorentz force acting on individual ions in solution [19]. They calculated that the trajectories of copper and sulfate ions in a CuS solution were dramatically perturbed by a magnetic field, supporting their hypothesis that Lorentz effect imaging could detect action currents. However, Wijesinghe and Roth claim that Truong et al. used an incorrect value of the mobility of these ions in their calculation [20]. When they used a more realistic value, the ion trajectories were not perturbed (Figure 7). Truong, working with Navid Pourtaheri and Craig Henriquez, have suggested that magnetohydrodynamic effects may explain the contrast obtained during Lorentz effect imaging [21,22]. This remains an open question.

## 4. MRI Measurement of Activity in the Brain

### 4.1. The Magnetic Field Produced by the Brain: Phantom Studies

Most researchers using MRI to sense biological currents want to monitor the brain. Several groups have tested the feasibility of this technique by recording from phantoms, in which current flowed into a container filled with saline [23,24,25,26,27]. Manbir Singh applied current through electrodes in a phantom, with a strength similar to the current associated with brain activity, during the 50 ms between the 90° and 180° radiofrequency pulses that were part of an MRI spin-echo pulse sequence. He concluded that measurement of action currents would require detecting a phase shift of about a third of a degree [28]. Bodurka and Bandettini performed similar experiments and concluded that a magnetic field as small as 0.2 nT lasting for 40 ms could be detected using MRI [29]. 

### 4.2. The Magnetic Field Produced by the Brain: Calculations

Several investigators have calculated the magnitude of the magnetic field created by brain activity [30,31,32,33,34]. For instance, Qingfei Luo et al. calculated the biomagnetic field specifically for the human brain [35]. They concluded that evoked signals are too small to be measured (the predicted phase shift was on the order of 0.02°), but that spontaneous activity may lead to larger signals (0.2° phase shift) that may be detectable. 

William Jay and his colleagues have calculated the magnetic field in the brain produced by currents in the neuron’s dendrites (minute projections from the cell body that couple to adjacent neurons) [36]. They found that a million uniformly oriented, simultaneously active dendrites could produce a magnetic field of nearly 8 nT and a phase shift of about 1°. They warn, however, that so many simultaneously active neurons would be unlikely in the brain, except perhaps during an epileptic seizure. If they use fewer dipoles that are randomly oriented, their magnetic field was less than a nanotesla and the phase shift was less than a tenth of a degree. Jay et al. speculate that, if anything, their calculations overestimate the strength of the magnetic field that the neurons would produce.

Dendritic signals have the advantage that they generally are not propagated and thus do not necessarily have both depolarization and repolarization phases that might cancel. The monophasic magnetic signal from dendrites has a much better chance of producing a net phase shift than the biphasic signal from a peripheral nerve.

**Figure 8:**
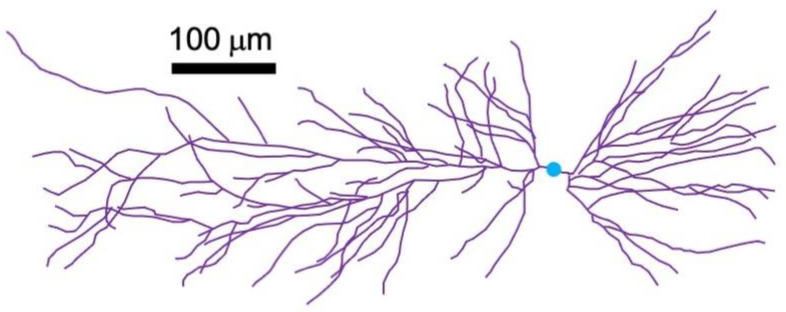
A hippocampal CA1 pyramidal cell used to model a neuron in the brain. The cell body is in blue and the dendrites are in purple. Adapted from Figure 2 of [37].

Antonino Cassara and his team performed even more detailed calculations of the MRI signal from the brain [37]. Their results depend on many factors, such as voxel size, neuron density, echo time, and the geometry of a neuron’s dendrites (Figure 8). Using reasonable parameters, they found phase shifts on the order of 0.0005°, which correspond to alterations in the MRI signal of 0.1 parts per million. The conclude that such changes “can probably not be detected by available MRI methodology” [37]. They also note that the magnetic field distribution is highly heterogeneous on a spatial scale similar to that of a neuron, and that the signal depends critically on the orientation of the magnetic field relative to the direction of the main static magnetic field of the MRI device. 

Ultra-low field MRI may be a particularly promising technique to detect neuronal current [38,39,40,41,42,43]. The Larmor frequency for hydrogen nuclei using an MRI device with a weak magnetic field is similar to the frequencies characteristic of neural activity, making resonant behavior more practicable. In other words, the neural magnetic field might be only one part in a billion compared with the static magnetic field of a 4 T MRI device, but the neural field might be as much as one part in a million compared with a 0.004 T low-field MRI device. Moreover, susceptibility effects, such as blood-oxygen-level-dependent signals, increase dramatically with magnetic field strength and will be negligible at ultra-low fields.

**Figure 9:**
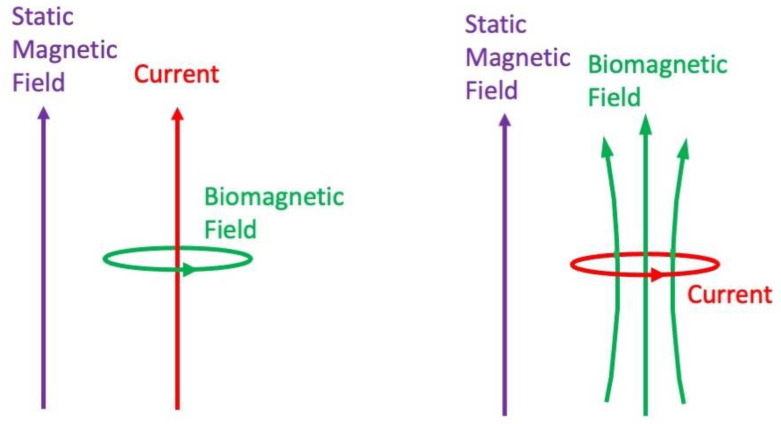
On the left, the current is in the same direction as the static magnetic field; the biomagnetic field has no component parallel to the static field. On the right, the current is in the plane perpendicular to the static magnetic field; the biomagnetic field has a component parallel to the static field. Only a biomagnetic field with a component parallel to the static magnetic field will change the phase of the spins.

Gisela Hagberg, Marta Bianciardi, and Bruno Maraviglia have examined the challenges of detecting neural activity using magnetic resonance imaging (which they refer to as “neural current-induced MRI” or “nc-MRI”) [44]. They suggest that postsynaptic potentials in dendrites will provide a more promising source of biocurrents than action potentials propagating along axons. They also assert that phase imaging will be more effective than magnitude imaging for this application. However, phase signals will compete with artifacts caused by motion from blood pulsations or breathing. MRI might be more sensitive if combined with electroencephalography or magnetoencephalography. Finally, currents flowing perpendicular to the static magnetic field of an MRI device should be easiest to detect (Figure 9).

### 4.3. The Magnetic Field Produced by the Brain: Detection

Several groups have attempted to detect the small magnetic fields of the brain using MRI. Some claim success [45,46,47,48,49,50,51] and others failure [28,52,53,54,55,56,57,58,59,60]. The key to a successful experiment is separating the signal caused by the direct detection of the magnetic field from the functional MRI signal caused by the blood-oxygen-level-dependent effect. Kamei et al. [45] attempted to remove effects arising from BOLD by subtracting signals obtained using opposite polarities of the read-out gradient and by subtracting rest images from activated images. Using this “double subtraction” method, they claimed to detect a neural signal caused by finger-tapping. Xiong et al. [46], Chow et al. [49], and Xue et al. [50] applied a stimulus at a high frequency (on the order of 1 Hz), which they claimed drove the slower BOLD response to steady state. These researchers also examined responses with short latencies following the stimulus (about 100 ms), so that the response was too fast to be caused by the BOLD mechanism. Bianciardi et al. [47] optimized their experiment for neural over BOLD response by using spin echo rather than gradient echo imaging and by synchronizing their pulse sequence to the stimulus optimally based on the timing of an independently measured electrical recording. Sundaram et al. [51] suppressed the BOLD signal by using a short repetition time of 47 ms. They studied interictal spikes associated with epilepsy and chose spikes with the proper timing by examining the simultaneously measured electroencephalogram. Petridou et al. [48] adopted a different strategy. They examined small rat-brain cell cultures that had no vascular system, eliminating the BOLD mechanism entirely. Each of these investigations reported a neural signal. However, each report noted that artifacts are possible, caused by other factors such as a fast component of the BOLD response, metabolically-driven temperature changes, or motion. 

Most investigators have studied magnetic fields evoked by a stimulus, but Daniel Konn and his collaborators have tried to measure the spontaneous alpha wave signal from the brain [61]. Alphas waves (8–12 Hz) produce the largest magnetic fields outside the brain, as measured by magnetoencephalography, and thus should produce a relatively big signal. However, Konn et al. did not observe any alpha waves [61]. Other teams have studied the magnetic field produced by the brain of a simpler animal, such as a snail [62] or an octopus [63].

### 4.4. Different Mechanisms Responsible for Brain Signals

**Figure 10:**
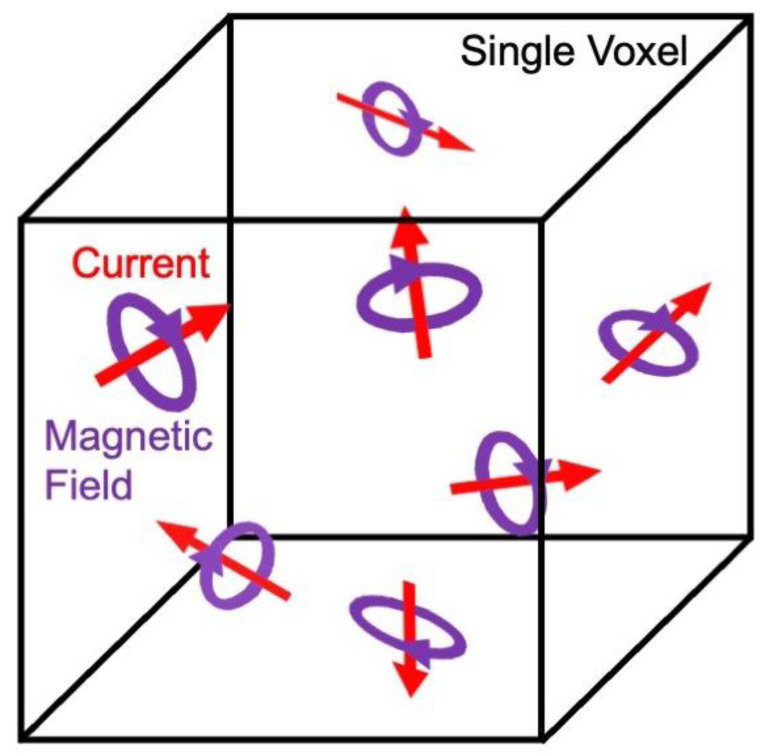
If the current (red) is heterogenous throughout a single voxel, the biomagnetic field (purple) will also be heterogenous. The resulting phase shift averaged over the voxel will be small, but the inhomogeneous biomagnetic field could cause the spins to dephase, shortening T_2_*.

While many researchers focus on phase shifts caused by neural magnetic fields, Lin Tang and coworkers note that these fields can also reduce the relaxation time constant T_2_* [55]. This would be expected if the neural magnetic field were produced by many neurons acting independently or in different directions, so their resultant magnetic field was heterogeneous (Figure 10). In that case, the different phase shifts experienced throughout a voxel would tend to dephase the spins, which is the same as the mechanism responsible for T_2_*. Changes in T_2_* would affect the magnitude of the MRI signal rather than its phase, and would in general reduce the MRI signal compared with when no neural activity is present. 

In 2012, Alan Koretsky explored a variety of mechanisms underlying changes in the MRI signal caused by neuronal activity [64]. Many of these mechanisms arise from factors other than the biomagnetic field produced by neurons. These mechanisms would compete with any signals resulting directly from magnetic field effects.

Koretsky calls imaging of electrical activity the “holy grail” for MRI, and these methods include not only the magnetic field effects discussed earlier, but also techniques such as finding molecules that are taken up by the neuron membrane and whose MRI signal depends on the transmembrane potential. Unfortunately, no such methods have been established yet. More promising appears to be contrast agents that can vary an MRI signal in response to calcium. For example, a molecule that contains known MRI contrast agents such as gadolinium or manganese [65] and that is sensitive to the intracellular calcium concentration could in principle vary the T_1_ relaxation time constant of MRI. Because intracellular calcium is often modulated by neuronal activity, this could provide a mechanism for MRI brain monitoring. Another approach would be to develop molecules whose MRI signal is modulated by gene expression. Koretsky reviews the extensive literature dealing with these efforts to develop molecular approaches for functional MRI [64].

Yet another possible source of image contrast is cell swelling. Denis Le Bihan and his coworkers have monitored diffusion using MRI and found changes correlated to neuronal activity [66]. They hypothesize that, when neurons are active, the relative size of a rapidly diffusing free water pool can decrease compared with the size of a more slowly diffusing water pool near the neuronal membrane. They speculate that such changes might be related to cell swelling during an action potential. However, Karla Miller et al. [67] claim that these signals are related to vascular blood flow rather than diffusion. Daniel Nunes and his associates found that diffusion MRI could monitor neural dynamics in rat experiments [68]. Several groups have investigated diffusion effects further [69,70]. For instance, Ruiliang Bai and coworkers, working in Peter Basser’s laboratory at the National Institutes of Health, simultaneously measured diffusion using MRI and calcium using fluorescence and found that the two techniques are highly correlated during neuronal hyperexcitability [71]. They conclude that the method may be useful for monitoring highly excitable states such as epilepsy, but that it does not appear to be sensitive enough to monitor normal brain activity.

## 5. Recent Experimental Results

Three recent studies have shed new light on the idea that MRI can be used to sense neural activity.

### 5.1. Sundaram et al. (2016)

**Figure 11:**
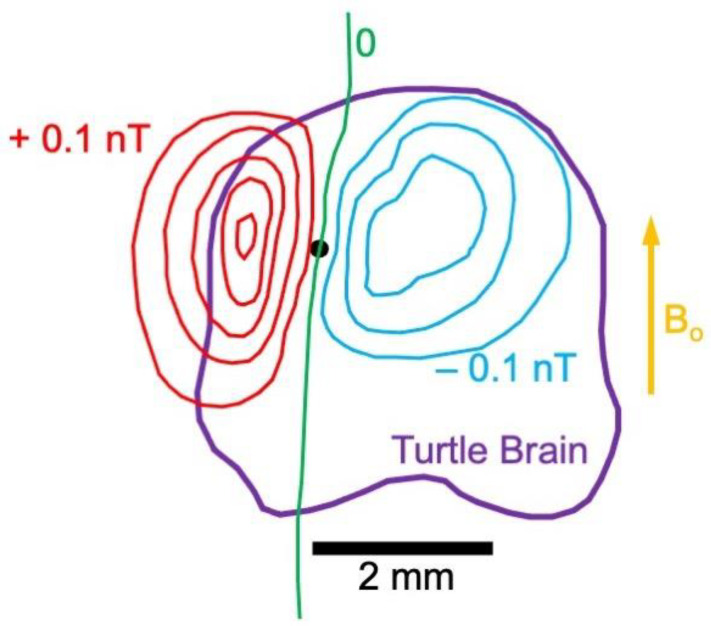
The magnetic field produced by a turtle cerebellum. The component of the biomagnetic field parallel to the static magnetic field of the MRI scanner (B_o_, yellow) is shown in a contour plot. Each contour corresponds to an increment of 0.1 nT; red contours mean the biomagnetic field is parallel to B_o_ (positive) and blue contours mean it is opposite to B_o_ (negative); the zero contour is green. The black dot indicates the approximate position of a current dipole directed into the paper. Adapted from Figure 10A in [72].

An article by Padmavathi Sundaram and her collaborators, coming out of Yoshio Okada’s laboratory at Boston Children’s Hospital, provides what may be the best evidence yet for MRI sensing of bioelectric currents [72]. They recorded from an intact isolated whole cerebellum of a turtle (Figure 11). This preparation was used because it can withstand hypoxia; the normal neuronal circuits are intact; the MRI signal is not contaminated by artifacts from blood flow, respiration, or motion; and it has a flat geometry that facilitates mapping experiments. Slow local field potentials were mediated by metabotropic glutamate receptors. Sundaram et al. showed that the signal originated from neurons by blocking it with kynurenic acid, a non-specific blocker of excitatory amino acid receptors. Likewise, they showed that the signal arose from glutamate receptors using the glutamate receptor blocker LY341495. Before performing experiments on the cerebellum, they calibrated their 4.7 T MRI machine using a phantom containing copper sulfate and carrying a current of 100 microamps.

Electrical activity was elicited using a 5 milliamp, 0.1 millisecond constant current stimulus. One strength of Sundaram et al.’s study is that it measured both the MRI signal and the simultaneous extracellular electrical voltage, thereby ensuring that the magnetic resonance phase change happened at the same time as the electrical potential. In some experiments, the electrical potential was mapped throughout the cerebellum.

Sundaram et al. were able to demonstrate that the phase shift is time-locked to the recorded electrical potential and their temporal waveforms matched. The phase shift, typically on the order of a few tenths of a degree, corresponds to a magnetic field strength of nearly a nanotesla. The distribution of the magnetic field had a dipole-like pattern (Figure 11) with regions of both positive and negative field, again consistent with the measured potential. Phase shifts were recorded with a 100 ms temporal resolution. Because the preparation was dissected from the animal and perfused by saline, there was no blood flow, so no chance of contamination by the blood-oxygen-level-dependent mechanism. Sundaram et al.’s careful study has few interpretations other than direct MRI detection of neuronal currents. Although the preparation is quite different than recording from the living human cortex, it nevertheless establishes in principle the possibility of using MRI as a sensor of brain activity.

### 5.2. Truong et al. (2019)

Trong-Kha Truong and his colleagues, working in the laboratory of Allen Song at Duke University, developed a new MRI imaging method to record neural activity in humans [73]. The key to their method is to use the spin-lock technique [41,42,43,74,75,76,77,78]. 

**Figure 12:**
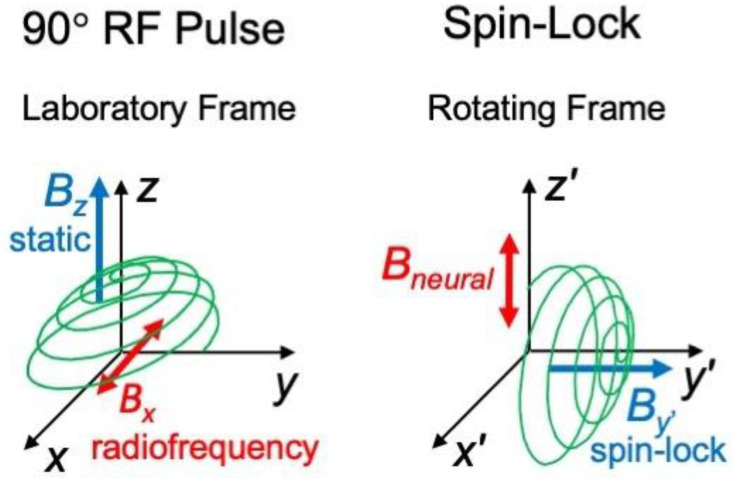
Comparison of a 90° radiofrequency pulse in a traditional MRI pulse sequence to a neural magnetic field in a spin-lock MRI pulse sequence. In the traditional experiment (**left**), the radiofrequency pulse rotates the spins from the direction of the static field (*z*) into the transverse (*x*-*y*) plane. In a spin-lock experiment (**right**), the neural magnetic field rotates the spins from the *y*′ direction in the rotating frame into the *x*′-*z*′ plane.

In traditional MRI, initially, the spins are aligned with the static magnetic field of the magnetic resonance imaging device (usually called the *z* direction). To excite the spins, you apply a radiofrequency magnetic field in the *x* direction oscillating at the Larmor frequency. Because this field is in resonance with the spin’s precession frequency, it rotates the spins from the *z* direction into the *x*-*y* plane (Figure 12, left; the tip of the spins follow the green path viewed from the laboratory frame of reference). If you turn this field off at just the right time, the spins end up all in the *y* direction and you have produced what is called a 90° pulse. The spins will then precess about the *z* axis at the Larmor frequency, remaining in the *x*-*y* plane until they finally relax back toward equilibrium.

In a spin-lock experiment, after an initial 90° radiofrequency pulse, a transverse magnetic field is applied, oscillating at the Larmor frequency, that is *circularly polarized*. In a frame of reference rotating at the Larmor frequency (*x*′-*y*′), this magnetic field appears stationary along the *y*′ axis, in the same direction as the spins (it is “spin-locked”). Now, the situation viewed from the rotating frame is similar to the starting point of a traditional pulse sequence viewed from the laboratory frame; there exists a static magnetic field with spins aligned with it. In the spin-lock case, the spins will precess about the *y*′ axis, but at a frequency much lower than the Larmor frequency because the circularly polarized magnetic field is much weaker than the static field. 

What could cause these spins to rotate into the *x*′-*z*′ plane? If an oscillating neural field is present at the same frequency as the spin precession frequency, so they are in resonance, it will cause the spins to rotate from the *y*′ direction into the *x*′-*z*′ plane (Figure 12, right; green path). In this way, the neural magnetic field in a spin-lock experiment acts like the radiofrequency excitation pulse in a traditional MRI experiment. If you turn off the circularly polarized magnetic field at just the right time, you have spins aligned in the *z*′ direction, ready for a traditional pulse sequence. However, if the spins do not experience the neural field, they remain in the *y*′ direction, with no component in the *z*′ direction, until they finally relax. 

Following this spin-lock pulse sequence, you can immediately perform traditional spin-echo or gradient-echo imaging. The experiment will proceed as normal, except that the initial amplitude of the spins in the *z* direction will depend on whether a neural magnetic field was present. Thus, the neural field will serve as a contrast agent for the image. This exquisitely sensitive method operates analogously to a lock-in amplifier, focusing on a particular frequency in the neural signal.

Truong et al. applied this technique to healthy human volunteers undergoing an eyes-open/eyes-closed task. The spin-lock frequency was set to match brain activity of alpha waves (8–12 Hz). Preliminary phantom studies suggest that the method can detect magnetic field oscillations as small as 0.06 nT. Several control experiments were performed to eliminate artifacts caused by the blood-oxygen-level-dependent effect or other confounding factors. Truong et al. concluded that their experiments “provide evidence suggesting that this new technique has the potential to noninvasively and directly image neuroelectric activity in the human brain in vivo” [73].

### 5.3. Toi et al. (2022)

One of the most recent papers highlighted in this review is by Phan Tan Toi and his coworkers at Sungkyunkwan University in South Korea [79]. They adopted an approach that allows millisecond temporal resolution using MRI, while not sacrificing spatial resolution. Their experiment uses a two-dimensional gradient-echo imaging sequence and a 9.4 T MRI device. The echo time and repetition time were reduced to only a few milliseconds by swapping the order of the repetition and phase encoding loops in the pulse sequence. 

**Figure 13:**
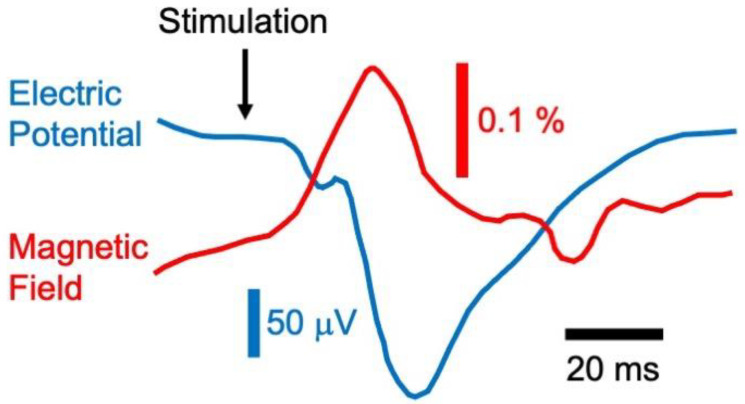
The magnetic field and the electrical potential measured by Toi et al. The electric and magnetic signals had similar latencies, about 25 ms, and thus may both be associated with the same neural source and are far too fast to be explained by the blood-oxygen-level-dependent mechanism of fMRI. Adapted from Figure 1I in [79].

Brain activity in mice was evoked using whisker-pad stimulation (Figure 13). The MRI signal increased by about 0.2% during stimulation. This change occurred around 25 ms after whisker-pad stimulation, which is too fast for a response owing to the blood-oxygen-level-dependent mechanism. The MRI signal had a latency that just about matched that of the simultaneously measured electrically recorded voltage. The experiment could capture the propagation of the neuronal activity along the thalamocortical pathway.

Toi et al. claim that their signal cannot be caused by the neuronal magnetic field, because the signal was increased, not decreased, by neural activity [79]. A biomagnetic field should produce phase shifts or changes in T_2_* that decrease the MRI signal (they did not use the spin-lock method, which could increase the magnetic resonance signal). The authors speculate that their signal may be due to an increase in T_2_ caused by the hydration of water near the membrane or by the cell swelling that accompanies neural depolarization. Thus, it seems that Toi et al.’s experiment is not an example of MRI sensing of neuronal magnetic fields directly, but it does highlight competing factors that will make measuring such magnetic fields difficult.

In an editorial accompanying Toi et al.’s article, Timo van Kerkoerle and Martijn Cloos conclude that “the ability of [direct imaging of neural activity] to lift the temporal and spatial hurdles that now limit BOLD fMRI holds the exciting potential to reveal the detailed computational mechanisms of mental processing at the fast pace at which it unfolds” [80].

Table 1 shows a comparison of the three experiments discussed in this section.

## 6. Artificial Intelligence

Artificial Intelligence is a technique using sophisticated computer software to analyze data. This method has not been applied yet to sensing of neural currents using MRI, as that is a new imaging modality that is not yet completely characterized. However, the closely related technique of fMRI, based on blood-oxygen-level-dependent contrast, has been analyzed extensively using artificial intelligence [81,82,83]. Serious medical problems such as Alzheimer’s disease and Schizophrenia have been studied using fMRI combined with artificial intelligence [82]. Most MRI technologists are excited about using artificial intelligence to improve medical imaging [84]. Thus, it may be a matter of time before artificial intelligence methods are applied to MRI sensing of neural currents.

Compressed sensing is one type of artificial intelligence, or “deep learning”, applied to fMRI data. Most MRI measurements extract data in the spatial frequency domain (“k-space”). One way to speed up an MRI scan is to undersample k-space and then fill in the missing data by extrapolation [85]. Artificial neural networks have been used to perform such compressed sensing. These networks typically consist of an input layer of data, followed by “hidden layers” of nodes, and then finally an output layer. Neural networks need to be trained by applying them to a data set. This training can be supervised if “ground truth” data are available (for instance, MRI data in which k-space was completely sampled and then undersampled before applying the neural network) or unsupervised if ground truth data are not available (for example, MRI data in which only undersampled k-space data were measured, so there is nothing to compare to the output of the neural network). Faster magnetic resonance imaging is critical. Often, high temporal and spatial resolution is required to detect neural currents, implying that obtaining the image takes a long time. Not only is this inconvenient and expensive, but it also makes the data more susceptible to motion artifacts as it is difficult to have a patient lie still for so long.

One challenge of using neural networks on MRI data is that you often need an enormous set of training data. For instance, when studying visual recognition tasks using fMRI, Emily Allen and her team have created a “Natural Scenes Dataset” consisting of tens of thousands of scenes [86]. Such data sets will need to be created before artificial intelligence can be used reliably to analyze neural activity.

For scientists, identifying and clarifying the detailed mechanisms underlying the measurement of neural currents using MRI is vital. From the point of view of medical doctors trying to use this technique to detect or diagnose disease, however, artificial intelligence methods may not require a complete understanding of fundamental mechanisms.

## 7. Conclusions

Whether or not J. H. Nagel actually measured neural activity using magnetic resonance imaging in 1984, he certainly did have a profound insight into a possible new technique for sensing brain activity. Recent experiments suggest that this technique is no fantasy and may ultimately become a reality. When coupled with modern methods such as artificial intelligence, monitoring neural activity using MRI may someday provide new information about how we think.

## Figures and Tables

**Table 1:** Comparison of three experiments to measure neural currents using MRI.

Reference	Sundaram et al. (2016) [72]	Truong et al. (2018) [73]	Toi et al. (2022) [79]
Field strength (T)	4.7	3	9.4
Preparation	Isolated turtle cerebellum	Normal human volunteer	Mouse
Pulse sequence	Gradient echo/Echo planar imaging	Spin-lock	2D gradient echo
Temporal resolution (ms)	100	125 spin-lock duration 500 repetition time	5
Spatial resolution (mm)	2.8 × 2.8 × 3	3.75 × 3.75 × 8	0.22 × 0.22 × 1
Stimulation protocol	Electrical stimulation using electrode	Eyes open/eyes close task, Alpha wave	Whisker-pad stimulation
Compare to electrical signal	Yes	No	Yes
Primarily sensitive to magnitude or phase	Phase	Magnitude	Magnitude
Method to eliminate BOLD	Bloodless preparation	Measurement with and without spin-lock, and other control experiments	Compare to conventional BOLD fMRI, use of a short echo time, and controls in reduced oxygen atmosphere

## Data Availability

Not applicable.

## References

[1] Nagel J.H. (1984). NMR imaging of action currents. IEEE Trans. Biomed. Eng..

[2] Joy M., Scott G., Henkelman M. (1989). In vivo detection of applied electric currents by magnetic resonance imaging. Magn. Reson. Imaging.

[3] Scott G.C., Joy M.L.G., Armstrong R.L., Henkelman R.M. (1991). Measurement of nonuniform current density by magnetic resonance. IEEE Trans. Med. Imaging.

[4] Scott G.C., Joy M.L.G., Armstrong R.L., Henkelman R.M. (1992). Sensitivity of magnetic-resonance current-density imaging. J. Magn. Reson..

[5] Ogawa S., Lee T.M., Kay A.R., Tank D.W. (1990). Brain magnetic resonance imaging with contrast dependent on blood oxygenation. Proc. Natl. Acad. Sci. USA.

[6] Kwong K.K., Belliveau J.W., Chesler D.A., Goldberg I.E., Weisskoff R.M., Poncelet B.P., Kennedy D.N., Hoppel B.E., Cohen M.S., Turner R. (1992). Dynamic magnetic resonance imaging of human brain activity during primary sensory stimulation. Proc. Natl. Acad. Sci. USA.

[7] Bandettini P.A., Petridou N., Boduraka J. (2005). Direct detection of neuronal activity with MRI: Fantasy, possibility, or reality?. Appl. Magn. Reson..

[8] Xu D., Roth B.J. (2017). The magnetic field produced by the heart and its influence on MRI. Math. Probl. Eng..

[9] Wikswo J.P., Barach J.P., Freeman J.A. (1980). Magnetic field of a nerve impulse: First measurements. Science.

[10] Roth B.J., Wikswo J.P. (1985). The magnetic field of a single axon: A comparison of theory and experiment. Biophys. J..

[11] Wijesinghe R.S., Roth B.J. (2009). Detection of peripheral nerve and skeletal muscle action currents using magnetic resonance imaging. Ann. Biomed. Eng..

[12] Paley M.N.J., Chow L.S., Whitby E.H., Cook G.G. (2009). Modeling of axonal fields in the optic nerve for direct MR detection studies. Image Vis. Comput..

[13] Yang H., Cook G.G., Paley M.N.J. (2003). Mapping of periodic waveforms using the ghost reconstructed alternating current estimation (GRACE) magnetic resonance imaging technique. Magn. Reson. Med..

[14] Song A.W., Takahashi A.M. (2001). Lorentz effect imaging. Magn. Reson. Imaging.

[15] Truong T.-K., Wilbur J.L., Song A.W. (2006). Synchronized detection of minute electrical currents with MRI using Lorentz effect imaging. J. Magn. Reson..

[16] Truong T.-K., Song A.W. (2006). Finding neuroelectric activity under magnetic field oscillations (NAMO) with magnetic resonance imaging in vivo. Proc. Natl. Acad. Sci. USA.

[17] Roth B.J., Basser P.J. (2009). Mechanical model of neural tissue displacement during Lorentz effect imaging. Magn. Reson. Med..

[18] Roth B.J., Luterek A., Puwal S. (2014). The movement of a nerve in a magnetic field: Application to MRI Lorentz effect imaging. Med. Biol. Eng. Comput..

[19] Truong T.-K., Avram A., Song A.W. (2008). Lorentz effect imaging of ionic currents in solution. J. Magn. Reson..

[20] Wijesinghe R.S., Roth B.J. (2010). Lorentz effect imaging of ionic currents in solution using correct values for ion mobility. J. Magn. Reson..

[21] Pourtaheri N., Truong T.-K., Henriquez C.S. (2013). Electromagnetohydrodynamic modeling of Lorentz effect imaging. J. Magn. Reson..

[22] Balasubramanian M., Mulkern R.V., Wells W.M., Sundaram P., Orbach D.B. (2015). Magnetic resonance imaging of ionic currents in solution: The effect of magnetohydrodynamic flow. Magn. Reson. Med..

[23] Bodurka J., Jesmanowicz A., Hyde J.S., Xu H., Estkowski L., Li S.-J. (1999). Current-induced magnetic resonance phase imaging. J. Mang. Reson..

[24] Konn D., Gowland P., Bowtell R. (2003). MRI detection of weak magnetic fields due to an extended current dipole in a conduction sphere: A model for direct detection of neuronal currents in the brain. Magn. Reson. Med..

[25] Pell G.S., Abbott D.F., Fleming S.W., Prichard J.W., Jackson G.D. (2006). Further steps toward direct magnetic resonance (MR) imaging detection of neural action currents: Optimization of MR sensitivity to transient and weak currents in a conductor. Magn. Reson. Med..

[26] Buracas G.T., Liu T.T., Buxton R.B., Frank L.R., Wong E.C. (2008). Imaging periodic currents using alternating balanced steady-state free precession. Magn. Reson. Med..

[27] Kim K.H., Heo H.-I., Park S.-H. (2018). Detection of fast oscillating magnetic fields using dynamic multiple TR imaging and Fourier analysis. PLoS ONE.

[28] Singh M. (1994). Sensitivity of MR phase shift to detect evoked neuromagnetic fields inside the head. IEEE Trans. Nucl. Sci..

[29] Bodurka J., Bandettini P.A. (2002). Toward direct mapping of neuronal activity: MRI detection of ultraweak transient magnetic field changes. Magn. Reson. Med..

[30] Hatada T., Sekino M., Ueno S. (2005). Finite element method-based calculation of the theoretical limit of sensitivity for detecting weak magnetic fields in the human brain using magnetic-resonance imaging. J. Appl. Phys..

[31] Park T.S., Lee S.Y. (2007). Effects of neuronal magnetic fields on MRI: Numerical analysis with axon and dendrite models. Neuroimage.

[32] Blagoev K.B., Mihaila B., Travis B.J., Alexandrov L.B., Bishop A.R., Ranken D., Posse S., Gasparovic C., Mayer A., Aine C.J. (2007). Modelling the magnetic signature of neuronal tissue. Neuroimage.

[33] Heller L., Barrowes B.E., George J.S. (2009). Modeling direct effects of neural current on MRI. Hum. Brain Mapp..

[34] Huang Y.-L., Xiong H.-C., Yao D.-Z. (2010). Direct MRI detection of the neuronal magnetic field: The effect of the dendrite branch. Phys. Med. Biol..

[35] Luo Q., Jiang X., Chen B., Zhu Y., Gao J.-H. (2011). Modeling neuronal current MRI signal with human neuron. Magn. Reson. Med..

[36] Jay W.I., Wijesinghe R.S., Dolasinski B.D., Roth B.J. (2012). Is it possible to detect dendrite currents using presently available magnetic resonance imaging techniques?. Med. Biol. Eng. Comput..

[37] Cassara A.M., Hagberg G.E., Bianciardi M., Migliore M., Maraviglia B. (2008). Realistic simulations of neuronal activity: A contribution to the debate on direct detection of neuronal currents by MRI. Neuroimage.

[38] Kraus R.H., Volegov P., Matlachov A., Espy M. (2008). Toward direct neural current imaging by resonant mechanisms at ultra-low field. Neuroimage.

[39] Cassara A.M., Maraviglia B. (2008). Microscopic investigation of the resonant mechanism for the implementation of nc-MRI at ultra-low field MRI. Neuroimage.

[40] Hofner N., Albrecht H.-H., Cassara A.M., Curio G., Hartwig S., Haueisen J., Hilschenz I., Korber R., Martens S., Scheer H.-J. (2011). Are brain currents detectable by means of low-field NMR? A phantom study. Magn. Reson. Imaging.

[41] Sveinsson B., Koonjoo N., Zhu B., Witzel T., Rosen M.S. (2020). Detection of nanotesla AC magnetic fields using steady-state SIRS and ultra-low field MRI. J. Neural Eng..

[42] Ueda H., Ito Y., Oida T., Taniguchi Y., Kobayashi T. (2020). Detection of tiny oscillatory magnetic fields using low-field MRI: A combined phantom and simulation study. J. Magn. Reson..

[43] Ueda H., Ito Y., Oida T., Taniguchi Y., Kobayashi T. (2021). Magnetic resonance imaging simulation with spin-lock preparations to detect tiny oscillatory magnetic fields. J. Magn. Reson..

[44] Hagberg G.E., Bianciardi M., Maraviglia B. (2006). Challenges for detection of neuronal currents by MRI. Magn. Reson. Med..

[45] Kamei H., Iramina K., Yoshikawa K., Ueno S. (1999). Neuronal current distribution imaging using magnetic resonance. IEEE Trans. Magn..

[46] Xiong J., Fox P.T., Gao J.-H. (2003). Directly mapping magnetic field effects of neuronal activity by magnetic resonance imaging. Hum. Brain Mapp..

[47] Bianciardi M., Di Russo F., Aprile T., Maraviglia B., Hagberg G.E. (2004). Combination of BOLD-fMRI and VEP recordings for spin-echo MRI detection of primary magnetic effects caused by neuronal currents. Magn. Reson. Imaging.

[48] Petridou N., Plenz D., Silva A.C., Loew M., Bodurka J., Bandettini P.A. (2006). Direct magnetic resonance detection of neuronal electrical activity. Proc. Natl. Acad. Sci. USA.

[49] Chow L.S., Cook G.G., Whitby E., Paley M.N.J. (2006). Investigation of MR signal modulation due to magnetic fields from neuronal currents in the adult human optic nerve and visual cortex. Magn. Reson. Imaging.

[50] Xue Y., Chen X., Grabowski T., Xiong J. (2009). Direct MRI mapping of neuronal activity evoked by electrical stimulation of the median nerve at the right wrist. Magn. Reson. Med..

[51] Sundaram P., Wells W.M., Mulkern R.V., Bubrick E.J., Bromfield E.B., Munch M., Orbach D.B. (2010). Fast human brain magnetic resonance responses associated with epileptiform spikes. Magn. Reson. Med..

[52] Chu R., de Zwart J.A., van Gelderen P., Fukunaga M., Kellman P., Holroyd T., Duyn J.H. (2004). Hunting for neuronal currents: Absence of rapid MRI signal changes during visual-evoked response. Neuroimage.

[53] Mandelkow H., Halder P., Brandeis D., Soellinger M., de Zanche N., Luechinger R., Boesiger P. (2007). Heart beats brain: The problem of detecting alpha waves by neuronal current imaging in joint EEG-MRI experiments. Neuroimage.

[54] Parkes L.M., de Lange F.P., Fries P., Toni I., Norris D.G. (2007). Inability to directly detect magnetic field changes associated with neuronal activity. Magn. Reson. Med..

[55] Tang L., Avison M.J., Gatenby J.C., Gore J.C. (2008). Failure to direct detect magnetic field dephasing corresponding to ERP generation. Magn. Reson. Imaging.

[56] Luo Q., Lu H., Lu H., Senseman D., Worsley K., Yang Y., Gao J.-H. (2009). Physiologically evoked neuronal current MRI in a bloodless turtle brain: Detectable or not?. Neuroimage.

[57] Rodionov R., Siniatchkin M., Michel C.M., Liston A.D., Thornton R., Guye M., Carmichael D.W., Lemieux L. (2010). Looking for neuronal currents using MRI: An EEG-fMRI investigation of fast MR signal changes time-locked to frequent focal epileptic discharges. Neuroimage.

[58] Luo Q., Jiang X., Gao J.-H. (2011). Detection of neuronal current MRI in human without BOLD contamination. Magn. Reson. Med..

[59] Huang J. (2014). Detecting neuronal currents with MRI: A human study. Magn. Reson. Med..

[60] Huang J., Zhu D.C. (2015). Exploring human brain neuronal currents with phase MRI. Int. J. Imaging Syst. Technol..

[61] Konn D., Leach S., Gowland P., Bowtell R. (2004). Initial attempts at directly detecting alpha wave activity in the brain using MRI. Magn. Reson. Imaging.

[62] Park T.S., Lee S.Y., Park J.-H., Cho M.H., Lee S.Y. (2006). Observation of the fast response of a magnetic resonance signal to neuronal activity: A snail ganglia study. Physiol. Meas..

[63] Jiang X., Lu H., Shigeno S., Tan L.-H., Yang Y., Ragsdale C.W., Gao J.-H. (2014). Octopus visual system: A functional MRI model for detecting neuronal electric currents without a blood-oxygen-level-dependent confound. Magn. Reson. Med..

[64] Koretsky A.P. (2012). Is there a path beyond BOLD? Molecular imaging of brain function. Neuroimage.

[65] Barandov A., Bartelle B.B., Williamson C.G., Loucks E.S., Lippard S.J., Jasanoff A. (2019). Sensing intracellular calcium ions using a manganese-based MRI contrast agent. Nat. Commun..

[66] Le Bihan D., Urayama S.-I., Aso T., Hanakawa T., Fukuyama H. (2006). Direct and fast detection of neuronal activation in the human brain with diffusion MRI. Proc. Natl. Acad. Sci. USA.

[67] Miller K.L., Bulte D.P., Devlin H., Robson M.D., Wise R.G., Woolrich M.W., Jezzard P., Behrens T.E.J. (2007). Evidence for a vascular contribution to diffusion fMRI at high b value. Proc. Natl. Acad. Sci. USA.

[68] Nunes D., Gil R., Shemesh N. (2021). A rapid-onset diffusion functional MRI signal reflects neuromorphological coupling dynamics. Neuroimage.

[69] Tsurugizawa T., Ciobanu L., Le Bihan D. (2013). Water diffusion in brain cortex closely tracks underlying neuronal activity. Proc. Natl. Acad. Sci. USA.

[70] Williams R.J., Reutens D.C., Hocking J. (2016). Influence of BOLD contributions to diffusion fMRI activation of the visual cortex. Front. Neurosci..

[71] Bai R., Stewart C.V., Plenz D., Basser P.J. (2016). Assessing the sensitivity of diffusion MRI to detect neuronal activity directly. Proc. Natl. Acad. Sci. USA.

[72] Sundaram P., Nummenmaa A., Wells W., Orbach D., Orringer D., Mulkern R., Okada Y. (2016). Direct neural current imaging in an intact cerebellum with magnetic resonance imaging. Neuroimage.

[73] Truong T.-K., Roberts K.C., Woldorff M.G., Song A.W. (2019). Toward direct MRI of neuro-electro-magnetic oscillations in the human brain. Magn. Reson. Med..

[74] Witzel T., Lin F.-H., Rosen B.R., Wald L.L. (2008). Stimulus-induced rotary saturation (SIRS): A potential method for the detection of neuronal currents with MRI. Neuroimage.

[75] Halpern-Manners N.W., Bajaj V.S., Teisseyre T.Z., Pines A. (2010). Magnetic resonance imaging of oscillating electrical currents. Proc. Natl. Acad. Sci. USA.

[76] Jiang X., Sheng J., Li H., Chai Y., Zhou X., Wu B., Guo X., Gao J.-H. (2016). Detection of subnanotesla oscillatory magnetic fields using MRI. Magn. Reson. Med..

[77] Ito Y., Ueno M., Kobayashi T. (2020). Neural magnetic field dependent fMRI toward direct functional connectivity measurements: A phantom study. Sci. Rep..

[78] Unger D.M., Wiest R., Kiefer C., Raillard M., Dutil G.F., Stein V.M., Schweizer D. (2021). Neuronal current imaging: An experimental method to investigate electrical currents in dogs with idiopathic epilepsy. J. Vet. Intern. Med..

[79] Toi P.T., Jang H.J., Min K., Kim S.-P., Lee S.-K., Lee J., Kwag J., Park J.-Y. (2022). In vivo direct imaging of neuronal activity at high temporospatial resolution. Science.

[80] van Kerkoerle T., Cloos M.A. (2022). Creating a window into the mind. Science.

[81] Pereira F., Mitchell T., Botvinick M. (2009). Machine learning classifiers and fMRI: A tutorial overview. Neuroimage.

[82] Wen D., Wei Z., Zhou Y., Li G., Zhang X., Han W. (2018). Deep learning methods to process fMRI data and their application in the diagnosis of cognitive impairment: A brief overview and our opinion. Front. Neuroinform..

[83] Yin W., Li L., Wu F.-X. (2022). Deep learning for brain disorder diagnosis based on fMRI images. Neurocomputing.

[84] Abuzaid M.M., Tekin H.O., Reza M., Elhag I.R., Elshami W. (2021). Assessment of MRI technologists in acceptance and willingness to integrate artificial intelligence into practice. Radiography.

[85] Chen Y., Schonlieb C.-B., Lio P., Leiner T., Dragotti P.L., Wamg G., Rueckert D., Firmin D., Yang G. (2022). AI-based reconstruction for fast MRI: A systematic review and meta-analysis. Proc. IEEE.

[86] Allen E.J., St-Yves G., Wu Y., Breedlove J.L., Prince J.S., Dowdle L.T., Nau M., Caron B., Pestilli F., Charest I. (2022). A massive 7T fMRI dataset to bridge cognitive neuroscience and artificial intelligence. Nat. Neurosci..

